# Sleeping in Church

**Published:** 1859-07

**Authors:** 


### SLEEPING IN CHURCH.

This unseemly act arises from the want of interest in the services. The most effectual physical preventative is to take a short nap just before going to church. All know what a painful effort it requires to excite to wakefulness; that very effort prevents all efficient attention to the discourse. Of ihe two evils, sleeping at home, and sleeping at church, the former is the less, and is valuable, because it is a certain remedy, and will allow a wholesome, wakeful attention to a discourse of very moderate interest.

